# Dissemination of Information on Selective Serotonin Reuptake Inhibitors on TikTok: Analytical Mixed Methods Study of Creator Types, Content Tone, and User Engagement

**DOI:** 10.2196/77383

**Published:** 2025-09-30

**Authors:** Brittany Quinn, Lindsey Nichols, Jennifer Frazee, Mark Payton, Rachel M A Linger

**Affiliations:** 1 Department of Biomedical Sciences, College of Osteopathic Medicine, Rocky Vista University, 8401 S Chambers Rd, Englewood, CO, 80112, United States, 1 303 373 2008

**Keywords:** TikTok, SSRI, medical professional, antidepressant, selective serotonin reuptake inhibitor, social media, engagement, video tone

## Abstract

**Background:**

TikTok [ByteDance] is a significant source of mental health–related content, including discussions on selective serotonin reuptake inhibitors (SSRIs). While the app fosters community building, its algorithm also amplifies misinformation as influencers without relevant expertise often dominate conversations about SSRIs. These videos frequently highlight personal experiences, potentially overshadowing evidence-based information from health care professionals. Despite these concerns, TikTok holds potential as a tool for improving mental health literacy when used by professionals to provide credible information.

**Objective:**

This study aimed to examine TikTok videos on SSRIs, hypothesizing that content will predominantly emphasize negative experiences and that videos by nonmedical professionals will attract higher engagement. By analyzing creators, engagement metrics, content tone, and video tone, this study aimed to shed light on social media’s role in shaping perceptions of SSRIs and mental health literacy.

**Methods:**

A sample of 99 TikTok videos was collected on December 8, 2024. Apify, a web scraper, compiled pertinent engagement metrics (URLs, likes, comments, and shares). Views were manually recorded. In total, 3 researchers evaluated video and content tones and documented findings in Qualtrics. User profiles were analyzed to classify creators as a “medical professional” or “nonmedical professional” based on verification of their credentials. Statistical analyses evaluated the hypotheses.

**Results:**

The number of videos created by both nonmedical and medical professionals was roughly even. Approximately one-third (35/99, 35%) mentioned a specific SSRI (ie, fluoxetine, fluvoxamine, vilazodone, sertraline, paroxetine, citalopram, or escitalopram). Compared to medical professionals, nonmedical creators produced significantly more videos with a positive video tone (*P*<.001). TikToks made by both groups of creators, however, had negative content tones (*P*=.78). Nonmedical professionals received significantly greater overall views (*P*=.01), likes (*P*=.01), and comments (*P*=.03), but overall shares were not significantly different (*P*=.18). Daily interaction metrics revealed that nonmedical professionals received more daily interaction, but these differences were not significant in terms of views (*P*=.09), likes (*P*=.06), comments (*P*=.15), or shares (*P*=.28).

**Conclusions:**

Results showed that while both creator groups focused on negative SSRI side effects and experiences (content tone), the way they presented this information (video tone) differed. Medical professionals generally maintained a neutral video tone, whereas nonmedical professionals were more likely to adopt a positive video tone. This may explain why nonmedical professionals’ videos had significantly more cumulative views, likes, and comments than medical professionals’ videos. These findings are consistent with other research suggesting that the TikTok algorithm and users are more likely to favor and engage with videos that evoke a strong emotional response and are perceived as relatable to viewers. This study highlights the need for medical professionals to improve their approach to content creation on TikTok by using a more positive video tone to increase engagement.

## Introduction

TikTok, a social media platform with over 1 billion active users, has become a hub for mental health-related content. The app’s algorithm curates a personalized “For You” page based on user interaction, promoting videos that align with viewers’ interests. While this creates opportunities for community building and awareness, it also allows misinformation to flourish unchecked, raising significant concerns about the accuracy and reliability of the health information shared [1].

Among the most discussed topics on TikTok is the use of selective serotonin reuptake inhibitors (SSRIs), a class of antidepressant medications commonly prescribed for conditions like depression and anxiety. Young adults aged 12‐24 years, a demographic that heavily uses TikTok, often turn to the platform for information on SSRIs, including personal experiences, side effects, and treatment efficacy [2]. While this can reduce stigma and encourage individuals to seek help, it also exposes users to unverified claims and anecdotal evidence that may misrepresent these medications [1,3].

TikTok’s unregulated content creation environment exacerbates these concerns. The platform does not verify the accuracy of videos, and the “verified” status of influencers is based on popularity and engagement rather than scientific or medical expertise. As a result, influential content creators without medical training often post videos based on personal experiences or unsubstantiated claims. These posts can reach millions of viewers, potentially overshadowing evidence-based guidance offered by health care professionals [4]. While influencer videos can provide comfort and foster a sense of community, the lack of credible information can perpetuate misconceptions about SSRIs, leading to fear, distrust, or misuse of these medications [1,5].

Despite its risks, TikTok has shown promise as a tool for increasing mental health literacy when leveraged by professionals. Studies suggest that mental health professionals on TikTok can enhance public understanding of SSRIs, counter misinformation, and encourage users to seek appropriate care [2]. However, the discourse surrounding SSRIs on TikTok remains largely anecdotal, with limited research exploring how these discussions impact user perceptions and behaviors.

SSRIs are a cornerstone of treatment for mood and anxiety disorders, functioning by increasing the availability of serotonin in the brain to regulate mood. While generally safe and effective, SSRIs can cause side effects such as nausea, insomnia, and sexual dysfunction, adverse outcomes that often dominate discussions on social media platforms like TikTok. The nuanced nature of these medications demands accurate, balanced information to support informed decision-making by viewers and patients alike [6]. However, TikTok’s emotionally and socially engaging format presents a dual-edged sword: while it has the potential to amplify health education, it can just as easily propagate misinformation.

Emerging literature has shown that TikTok videos created by nonmedical professionals receive significantly more engagement than those made by licensed clinicians, particularly in the context of anxiety, attention-deficit hyperactivity disorder, and depression. The most widely engaged videos related to these conditions were typically informal, personal in tone, and rarely produced by credentialed professionals [6-8]. Likewise, several studies reported that peer-driven content overwhelmingly outperformed clinician-created content in terms of likes, shares, and comments, suggesting that audience engagement may be more closely tied to relatability and tone than to professional credibility [9,10].

These findings underscore the critical role that both video tone (eg, music, pacing, and visual style) and content tone (eg, emotional, humorous, and serious) play in shaping audience responses on TikTok. Yet, while previous studies have categorized content themes and identified disparities in engagement, few have conducted systematic comparisons of video and content tone across creator types or explored how these stylistic choices influence public perception, particularly regarding pharmacologic treatments like SSRIs. Our study seeks to fill this gap by evaluating the emotional and aesthetic tone used, with attention to how these elements may contribute to audience engagement.

The underrepresentation of emotional resonance in educational content represents a missed opportunity for medical professionals to enhance trust and engagement on platforms like TikTok. By analyzing SSRI-related TikToks through the dual lens of video and content tone, our study builds upon previous research by linking stylistic elements to viewer engagement and perception. In doing so, we aim to identify communication strategies that could bridge the gap between clinical credibility and audience appeal in social media health messaging.

While previous studies have used similar methodological approaches to explore content dissemination and virality, this study extends their work by incorporating a dual-coded analysis of both content and video tone. We hypothesize that TikTok videos about SSRIs will predominantly emphasize negative side effects and personal experiences, thereby exhibiting a negative content tone. Furthermore, we expect that videos produced by nonmedical professionals will receive significantly higher engagement and will more frequently use a positive video tone, such as the use of trending songs, dance, and humor, compared to those made by health care professionals. By examining creator identity, audience engagement, and tonal presentation, this study aimed to contribute to the growing literature on digital health communication and deepen our understanding of how social media platforms shape mental health literacy, particularly in the context of pharmacologic treatment.

## Methods

### Overview

Before data collection, a test run was conducted using approximately 25 videos on TikTok to develop definitions for both video tone and content tone. This test run was conducted using a preexisting TikTok account, as the primary goal was to develop tone definitions rather than control for algorithmic bias. The sample consisted of the first 25 videos retrieved by the TikTok algorithm. A basic understanding of the differences between the overall video tone and the content tone of each video was put together via a Google search.

While video and content tone seem similar on the surface, there are distinct differences that contribute to the overall impression of each video. Video tone refers to the overall emotional and aesthetic design of the video and includes things such as visuals (lighting and color grading), music and sound design (dramatic music vs ambient noise), pacing (fast vs slow shots), and narration styles (warm, serious, quirky, etc). Content tone refers to the emotional or rhetorical attitude conveyed by the words themselves. Common content tones include informative, persuasive, humorous, and formal. The differences between content and video tone are exemplified by the examples, like the delivery of a video might feel lighthearted and humorous thanks to the use of a trending song or dance, while the content portrays a very different message and addresses serious topics such as depression or suicidal ideation. We further categorized content and video tones into being positive, negative, ambiguous, or neutral after noting pertinent themes and recurrent characteristics of the trial videos (Table 1). These defining criteria were used as a guide during data collection and analysis. After discussion with a statistician at Rocky Vista University regarding testing the hypotheses, a goal of approximately 100 TikTok videos was set to allow for adequate statistical analysis. A sample of 209 videos was collected on December 8, 2024, using the search term “SSRI.” The videos in the sample were the first 209 retrieved by the TikTok algorithm using a newly created account to avoid algorithm bias. Videos from the trial run were not automatically excluded if they were also retrieved by the search in the study’s newly created account. When a video appeared in both the trial run and the study sample, three independent reviewers re-evaluated it for video and content tone during the coding process. Out of the 209 URLs that were pulled, 129 videos were analyzed, 30 were excluded, and 99 were included in statistical analysis. Videos were excluded if they were unrelated to SSRIs (24 videos), were duplicates (5 videos), or were not in English (1 video). The remaining 80 videos were not analyzed because the desired sample size was achieved.

**Table 1.** Definitions of the four categories (positive, negative, neutral, and ambiguous) of video tone and content tone.

Tone	Overall video	Content
Positive	A TikTok video that exudes an uplifting, cheerful, and enthusiastic vibe. These videos are designed to evoke feelings of happiness, motivation, or amusement and contribute to the viewer feeling good and engaged. Examples include funny skits, heartwarming moments, or content that displays achievements and celebrations.	Mentions positive side effects including decreased anxiety, decreased depression, decreased panic attacks, improved suicidal thoughts or ideation, improved sleep, improved focus, decreased anger or irritability, improved social relationships, improved academic or work performance, desired weight gain or loss, or increased confidence. Can also mention improvement in the overall quality of one’s life, safety and effectiveness of the medication, or feelings of increased happiness.
Negative	A TikTok video that creates a sense of discomfort, sadness, or distress. These videos often have underlying themes of frustration or disappointment and are used to convey personal challenges or criticism. Videos with negative tones often include serious discussions, conflicts, or content that is meant to highlight problems, express anger, or bring awareness to difficult topics.	Mentions negative side effects including increased depression, anxiety, suicidal thoughts or ideation, psychosis, anger, insomnia, drowsiness, irritability, unwanted weight gain or loss, anhedonia, apathy, emotional blunting, nausea, vomiting, diarrhea, constipation, decreased libido, erectile dysfunction, fatigue, dry mouth, tremors, flushing, weakness, dizziness, headache, blurry vision, excessive sweating, dissociation, derealization, depersonalization, or mania. Can also mention decreased quality of life, ineffectiveness of medication, or experiences of withdrawal when missing a dose or discontinuing medication.
Ambiguous	A TikTok video that blends elements of positivity and negativity or does not clearly convey a single emotion. These videos may include content that is open to interpretation or mixes humor with underlying seriousness. Examples include content that might start as a playful trend but takes a surprising turn that leaves the viewer uncertain of how to feel. Videos with ambiguous tones can provoke thought, surprise, or intrigue by leaving the audience with mixed emotions or questions about the intention behind the video.	The video mentions both positive and negative symptoms or experiences.
Neutral	A TikTok video that does not include elements of positivity or negativity. Examples include videos that present facts without underlying bias.	The video does not mention positive or negative symptoms or experiences.

In total, 3 reviewers watched the videos in one afternoon and independently coded them in terms of video and content tone. Three coders were used to reduce individual bias and increase reliability. The reviewers then discussed their responses and came to a consensus and submitted a single Qualtrics survey, which was created to help consolidate the information from each video. The survey included the video URL, content creator’s name, date the video was published, overall video and content tones as agreed upon by all 3 evaluators, whether a specific SSRI was mentioned (if so, the specific medication was logged), if specific side effects of the drugs were mentioned (if so, there were positive and negative categories as well as a fill-in-the-blank option for less commonly mentioned side effects), and classifications of content creators as being either “nonmedical professionals” or “medical professionals.”

Apify, a cloud-based platform for web scraping and data extraction, was then used to quickly extract relevant video metrics for the study, including the cumulative number of comments, shares, and likes, as well as the creation date and URLs of each of the 99 videos previously identified. The information was exported into a Microsoft Excel spreadsheet. In a separate column on the spreadsheet, the coders manually logged the number of cumulative views each video received by December 8, 2024, the same day that the web scraper extracted the data, to limit discrepancies that timing could have caused. The cumulative views had to be manually logged because Apify did not have the capability to collect that information automatically. The use of a web scraper was favored over manual data entry due to its greater efficiency and improved accuracy. Each medical professional was verified on LinkedIn (Microsoft Corporation) or through institutional affiliations when possible. Unverified medical professionals were recorded under their claimed profession, with their verification status noted. The “medical professional” category was further subdivided into specific titles. Creators who did not align with any of the listed titles under “medical professional,” such as those with PhDs in pharmacy and neuroscience, or physician assistants, were categorized as “other” and counted as medical professionals. The decision to count these creators as medical professionals rather than nonmedical professionals was made due to their higher level of education in a field related to mental health.

The Qualtrics data was submitted to a statistician for analysis upon completion of data collection. All statistical analyses were performed using SAS (version 9.4; SAS Institute). Medical professionals were compared to nonmedical professionals with independent *t* tests for meaningfully numeric variables, such as the number of views or likes per day. Medical versus nonmedical videos were also compared using video tone or video content with the use of contingency tables and *χ*^2^ tests. Statistical significance was determined as *P*<.05.

### Ethical Considerations

This project was reviewed by the Rocky Vista University institutional review board on October 31, 2024, and was determined not to constitute human subjects research as defined by 45 code of federal regulations §46.102(e). The study involved the analysis of publicly available TikTok content and did not include user interaction, collection of direct identifiers, or efforts to determine the age of content creators. As such, institutional review board approval and informed consent were not required.

Only publicly accessible posts were analyzed, and no identifiable information, such as usernames, handles, profile images, or other details that could reasonably reveal the identity of individuals, especially minors, will be reported. Engagement metrics (eg, views, likes, comments, and shares) are presented solely in aggregate form.

All data collection was conducted in accordance with TikTok’s terms of service, and no scraping tools or automated extraction methods prohibited by the platform were used. Raw data are securely stored on access-controlled drives and will be retained in accordance with institutional data retention policies. The medical professional status of content creators was verified through publicly available sources, including LinkedIn profiles and institutional affiliations.

## Results

Nonmedical professionals created 53% (52/99) of the videos in this study, while medical professionals created the remaining 47% (47/99). Physicians (DOs/MDs) were responsible for 25% (25/99) of the videos, followed by pharmacists at 5% (5/99), neuroscientists at 4% (4/99), and nurse practitioners at 3% (3/99). Research scientists, naturopaths, and nurses each contributed 2% (2/99), while psychologists, therapists, medical assistants, and optometrists each accounted for 1% (1/99). Surprisingly, only 35% (35/99) of the videos mentioned a specific SSRI, while the remaining 64 videos, nearly two-thirds of the sample, did not.

**Figure 1.**
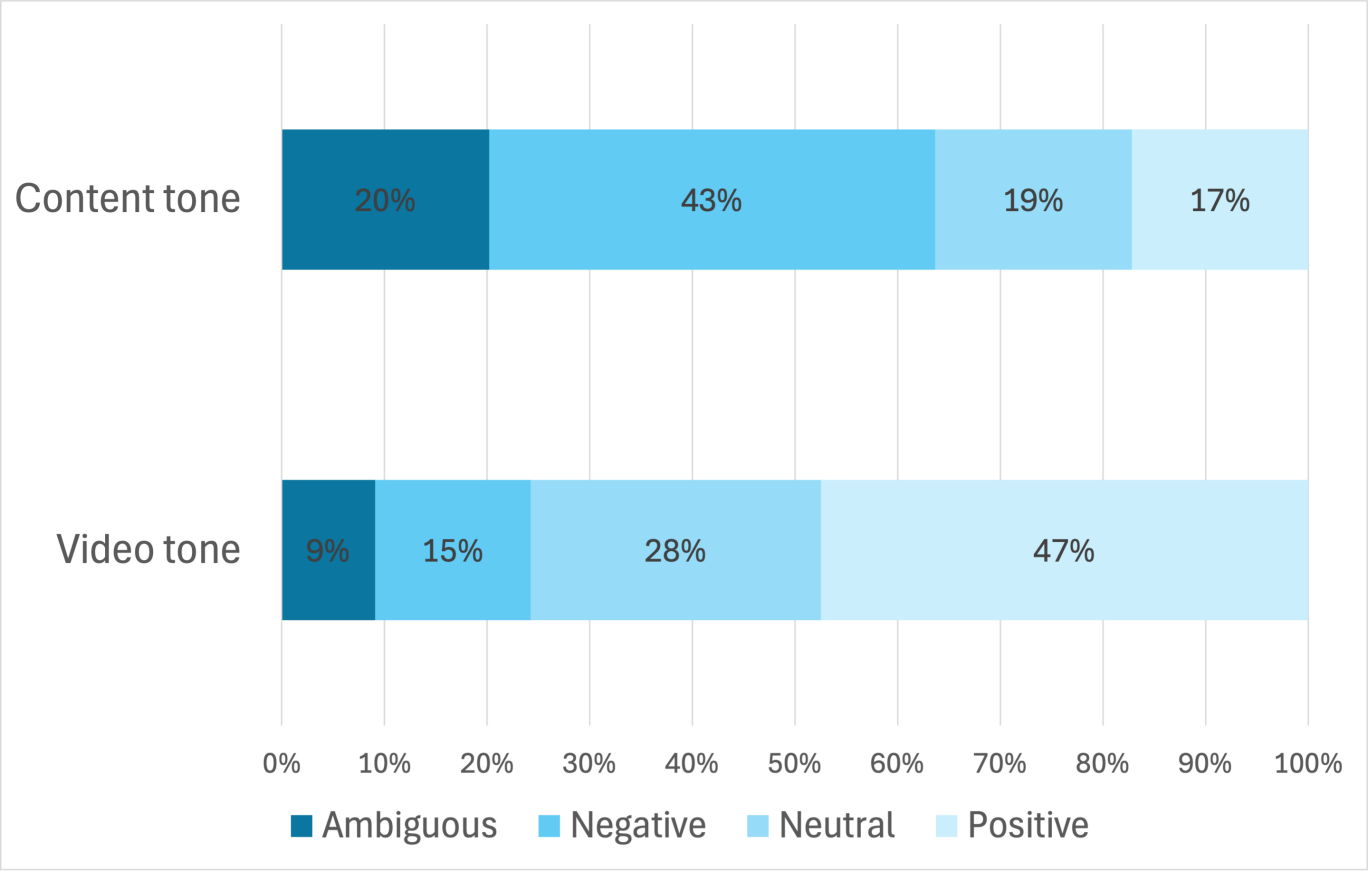
Stacked bar chart illustrating the overall differences between video and content tones in the sample. From left to right, the gradients of blue indicate either ambiguous, negative, neutral, or positive tones (n=99 videos).

The analysis revealed a notable discrepancy between video tone and content tone. While nearly half of the videos (46/99, 47%) conveyed a positive tone in their delivery, only 17% (16/99) presented content that was objectively positive. Conversely, although just 15% (14/99) of videos had an overtly negative tone, 43% (42/99) of the content was classified as negative (Figure 1). By aligning accurate, evidence-based information with the platform’s preferred styles of content delivery, providers can more effectively engage audiences and enhance public understanding of mental health treatment.

**Figure 2.**
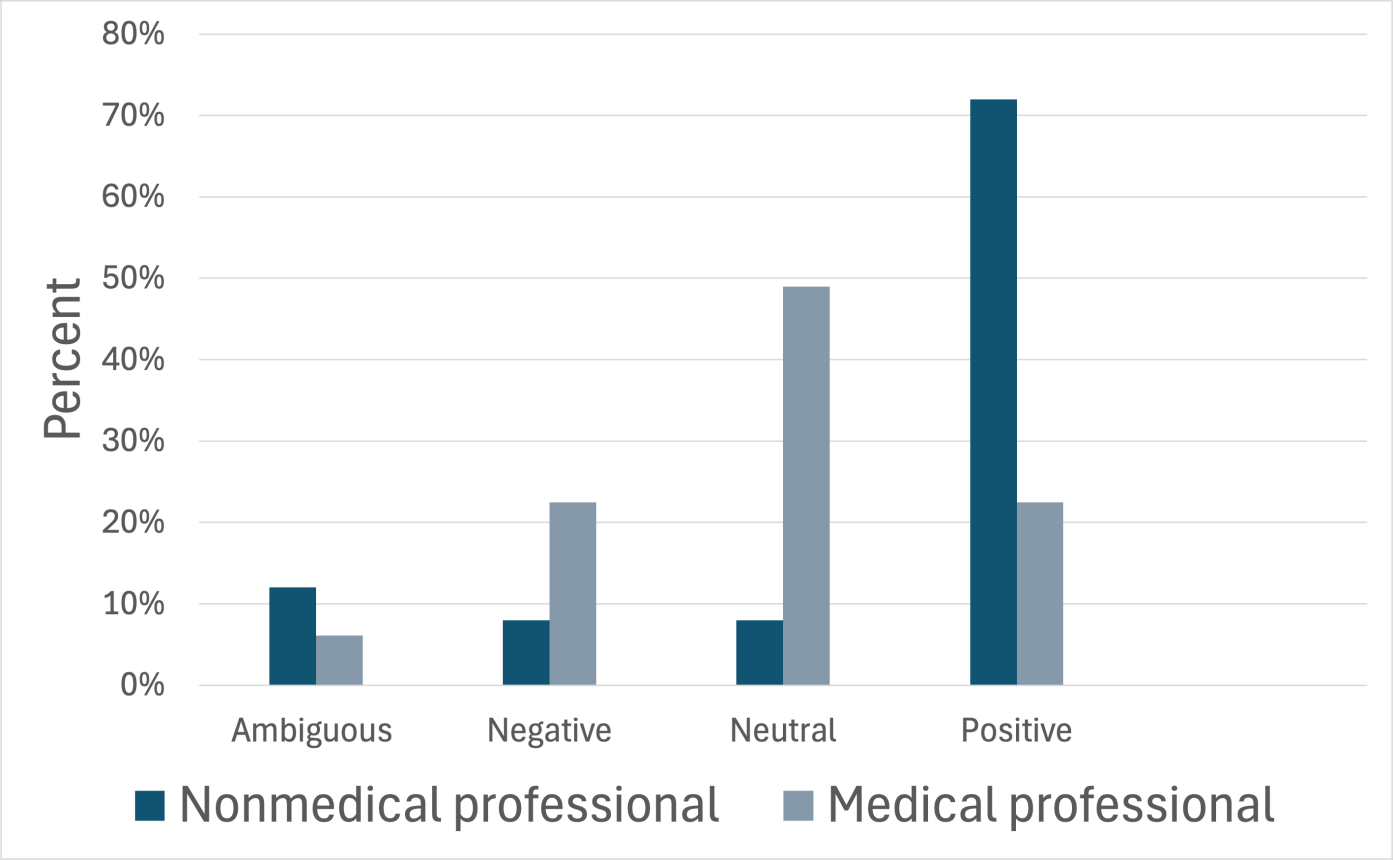
Comparison of the video tone between nonmedical professionals and medical professionals. The x-axis categorizes video tone as ambiguous, negative, neutral, or positive. The y-axis gives a percentage of videos (n=99) exhibiting each type of tone. Dark blue bars represent the percentage of videos produced by nonmedical professionals. Light blue-gray bars represent the percentage of videos created by medical professionals. A *χ*^2^ analysis returned an overall *P* value of <.0001.

**Figure 3.**
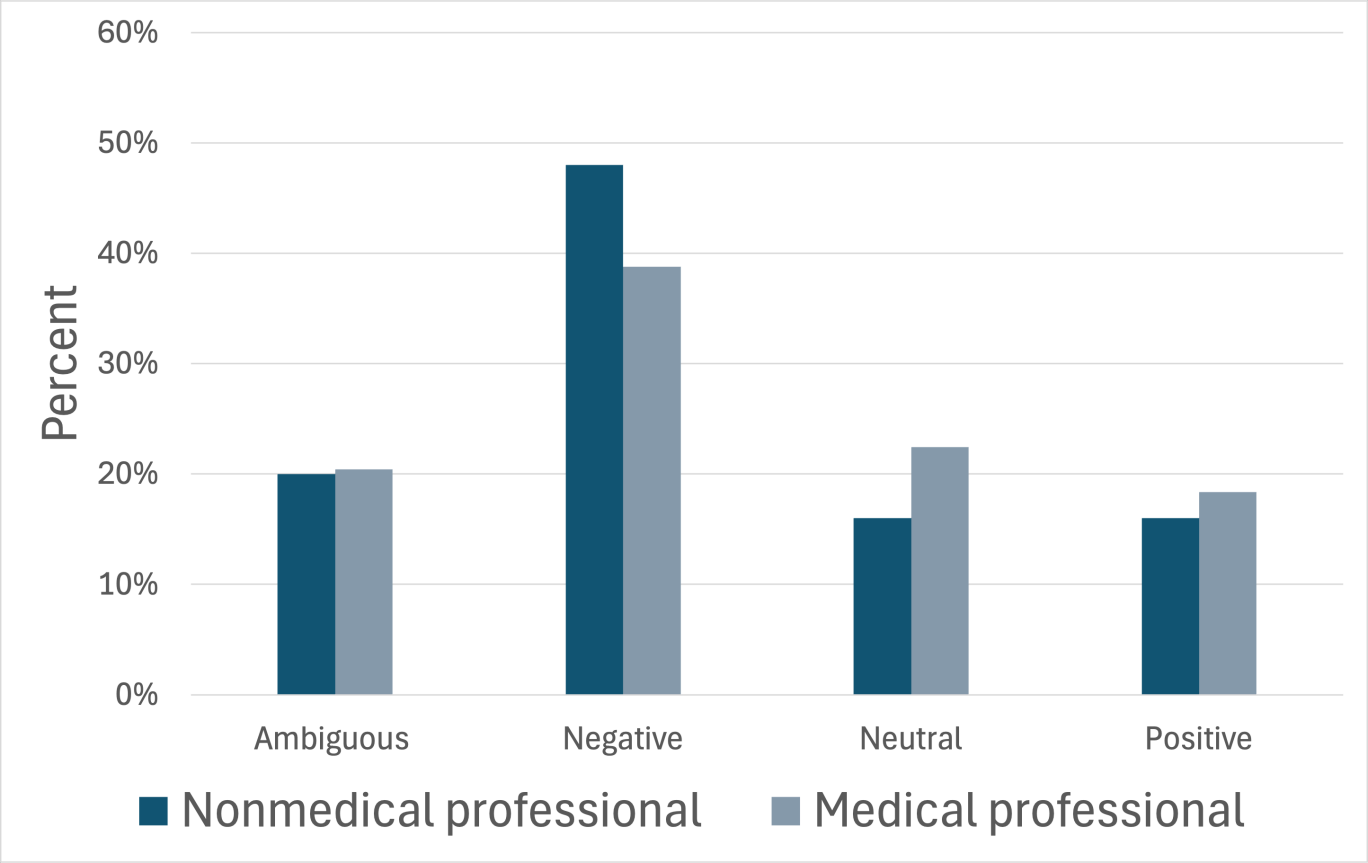
Comparison of the content tone between nonmedical professionals and medical professionals. The x-axis categorizes video tone as ambiguous, negative, neutral, or positive. The y-axis gives a percentage of videos (n=99) exhibiting each type of tone. Dark blue bars represent the percentage of videos produced by nonmedical professionals. Light blue-gray bars represent the percentage of videos created by medical professionals. A *χ*^2^ analysis returned an overall *P* value of .78, which is not statistically significant.

In agreement with our hypothesis, nonmedical professionals were found to have predominantly positive video tones (Figure 2). While differences in content tone did not meet our criteria for statistical significance, videos created by nonmedical professionals trended toward negative or ambiguous content tone (Figure 3). Medical professionals, on the other hand, had neutral video tone and negative or neutral content tones (Figures2  3). Notably, videos created by nonmedical professionals that often showcased a more positive video tone received significantly more cumulative engagement (Table 2). These results suggest a potential disconnect between presentation style and content being presented, particularly among nonmedical professionals.

**Table 2.** Engagement metrics for videos produced by nonmedical professionals and medical professionals. *P* values represent comparisons of medical versus nonmedical professionals for means of total views, views per day, total likes, likes per day, total comments, comments per day, total shares, and shares per day. Comparisons were made using a 2-sample *t* test.

Engagement metric	Nonmedical professionals	Medical professionals	*P* value
Total views	801,658.00	268,745.00	.01
Views per day	3382.70	1490.80	.09
Total likes	70,537.50	13,816.40	.01
Likes per day	298.60	80.03	.06
Total comments	866.50	289.90	.03
Comments per day	3.24	1.65	.15
Total shares	2775.60	1401.40	.18
Shares per day	14.50	7.50	.28

## Discussion

### Principal Results

We hypothesized that TikTok videos made by nonmedical professionals would receive significantly more engagement compared to those made by medical professionals. Our results partially supported this with videos made by nonmedical professionals receiving significantly more cumulative engagement. Daily engagement metrics tended to be higher for videos made by nonmedical professionals, but these differences were not statistically significant. This suggests that while videos from both groups are disseminated at similar rates, the TikTok algorithm may favor videos from nonmedical professionals, keeping them on users’ For You pages for an extended period, which could result in higher cumulative engagement metrics. This is consistent with findings showing that users find content from peers and influencers more engaging than content made by medical professionals [7,11], and medical professionals receive significantly less cumulative engagement on TikTok compared to nonmedical professionals [12,13].

This difference in engagement may be attributed to the fact that, although both groups predominantly created videos with a negative content tone, nonmedical professionals produced significantly more TikToks with a positive overall video tone, while medical professionals produced videos with neutral video tone. According to our definitions of content and video tones, this indicates that while both groups created a similar number of videos focusing on negative side effects or experiences with SSRIs, nonmedical professionals employed approaches such as humor, skits, and personal achievements to engage viewers and evoke positive emotions. This is consistent with research showing that the TikTok algorithm favors videos that evoke strong emotional responses, which may explain why neutral content created by medical professionals did not perform as well [14]. In addition to favoring videos with strong emotion, research has shown that videos that simultaneously used both humor and frustration received higher engagement, indicating that the presence of emotion in general, rather than a specific emotion, can lead to higher engagement. We suspect that medical professionals may have used a neutral tone to deliver unbiased information, but this approach likely reduced the emotional impact of their videos, making them seem authoritative and less relatable to viewers. This divergence suggests that the emotional framing of antidepressant-related videos may not reliably reflect the underlying message, underscoring the need for health care providers to better understand TikTok trends and leverage them strategically.

In addition to emotional appeal, studies indicate that engagement behaviors, such as liking and sharing, are often driven by perceived authenticity and relatability rather than reliability alone [9,15]. Medical professionals may be able to increase both their emotional appeal and authenticity by using trending sounds. Trending sounds on TikTok contribute to what are known as “affective audio networks,” where videos are connected through shared motifs across various contexts. This enables content creators to guide audiences toward specific emotional responses, thereby increasing engagement through shared emotional experiences [16]. In addition, while our study did not analyze the time of day each video was posted, previous research has shown that posting at specific times [2] can increase a video’s chances of trending and receiving higher engagement. This highlights the importance of overall video tone and content tone as an effective means of influencing communication on social media platforms and underscores an opportunity for medical professionals to adopt more engaging delivery strategies without compromising content accuracy.

### Limitations

This study’s findings are subject to several limitations. Creator credentials were verified through LinkedIn and institutional affiliations, which may not be completely accurate or up to date. Furthermore, we restricted the analysis to videos in English, which may exclude a significant subset of relevant content. The video coding was based on our pre-established tone definitions. To ensure the results were reliable, we used a method of triangulation: 3 researchers independently coded each video. They then discussed their coding decisions to reach a consensus, with the final tone categorization recorded in Qualtrics. Since a single, agreed-upon categorization was recorded for each video, we could not calculate a reliability percentage.

While this study examined the engagement metrics and content type produced by medical and nonmedical professionals, it did not examine the accuracy of information in the videos. Given the influence of social media on public perceptions of mental health and psychiatric medications, it is important for future studies to evaluate the accuracy of these TikToks. This will help determine whether a substantial amount of misinformation about SSRIs is being circulated on TikTok.

### Conclusion

In conclusion, this study highlights the impact of TikTok content and video tones on user engagement. While videos created by both medical professionals and nonmedical professionals focused on negative side effects and experiences with SSRIs, videos made by nonmedical professionals more often used a positive video tone, likely increasing engagement through appealing to emotions and appearing relatable. Given that nonmedical professionals had significantly more cumulative engagement, this study highlights the need for medical professionals to shift from using a neutral tone to a more positive tone to increase viewer engagement. Future studies can expand on these findings by evaluating the accuracy of SSRI-related content presented on TikTok by both groups and how that relates to tone.

## Abbreviations

SSRI: selective serotonin reuptake inhibitor
